# Smoking and its impact on eating behavior: A review

**DOI:** 10.6026/973206300220132

**Published:** 2026-01-31

**Authors:** Manoj Prabhakar, Kavitha Bottu, Preetha Elizabeth Chaly, Ramesh Kumar S.G, Ardhanaari M

**Affiliations:** 1 Department of Oral Pathology and Microbiology, Meenakshi Ammal Dental College and Hospital, Meenakshi Academy of Higher Education and Research, Chennai, Tamilnadu, India; 2 Department of Public Health Dentistry, Meenakshi Ammal Dental College and Hospital, Meenakshi Academy of Higher Education and Research, Chennai, Tamilnadu, India; 3 Department of Public Health Dentistry, Tamilnadu Government Dental College and Hospital, Chennai, Tamilnadu, India; 4 Department of Psychiatry, Meenakshi Medical College Hospital and Research Institute, Meenakshi Academy of Higher Education and Research, Enathur, Tamilnadu, India

**Keywords:** Smoking, eating behavior, dietary preferences

## Abstract

Smoking and its association with eating and dietary behaviors is complex, with smokers exhibiting varied dietary patterns. It is a widespread global deleterious behavior with significant effects on various physiological and psychological processes, including appetite regulation and dietary habits. Although the adverse health consequences of smoking are well recognized, its impact on eating behavior remains complex and less elucidated. Smokers often exhibit a preference for highly palatable, energy dense and ultra-processed foods due to a combination of physiological, neurochemical and psychological factors. Nicotine, the primary psychoactive component of tobacco, is known to cause appetite-suppressing effects leading many smokers to report reduced food intake on one hand and causing increased appetite leading to obesity on the other. This dual and contrasting biological mechanism contributes to differences in dietary preferences, food intake patterns and weight fluctuations observed among smokers. It is therefore essential to explore this intricate connection between smoking, appetite regulation and dietary habits and the factors associated with it.

## Background:

Smoking is a widespread behavior with significant effects on various physiological and psychological processes, including appetite regulation and eating habits. Tobacco use remains a major public health concern, as it is a leading cause of preventable diseases such as cancer, cardiovascular disease and respiratory disorders. While the detrimental health effects of smoking are well-established, its influence on eating behavior is a complex and less understood phenomenon [1]. Nicotine, the primary psychoactive component of tobacco, is known to have appetite- suppressing effects, leading many smokers to report reduced food intake and weight control as a motivation for smoking. Studies have shown that nicotine interacts with the brain's reward system and alters neurotransmitter activity, particularly dopamine and serotonin, which play key roles in hunger and satiety regulation [2, 3, 4, 5-6]. This biological mechanism contributes to differences in dietary preferences, food intake patterns and weight fluctuations observed among smokers. Moreover, smoking has been linked to changes in taste perception and food preferences. Literature suggests that smokers exhibit reduced sensitivity to sweet and bitter tastes, which may influence their consumption of highly palatable, energy-dense foods [7]. Smokers often consume beverages, sweets, or snacks immediately after smoking due to a combination of physiological, sensory and psychological factors. Additionally, smoking cessation often results in weight gain, further complicating the relationship between tobacco uses and eating behavior [8]. These changes pose challenges for individuals attempting to quit smoking, as concerns about weight gain can be a barrier to cessation efforts. Therefore, it is of interest to review on the physiological, neurochemical, psychological and behavioral aspects of smoking-related dietary changes, with a focus on the implications for public health and clinical interventions.

## Physiological mechanism linking smoking and eating behavior:

Smoking significantly influences eating behavior through various physiological mechanisms, primarily mediated by nicotine. These mechanisms include appetite suppression, alteration in metabolism and energy balance and impact on hunger-related hormones such as ghrelin and lepton [2, 9]. Nicotine, the primary addictive component in tobacco, has been identified as a potent appetite suppressant. Literature indicates that nicotine activates specific pathways in the brain that reduce hunger sensations. Nicotine also stimulates the hypothalamic melanocortin system, which plays a crucial role in regulating feeding behavior and energy homeostasis. This activation leads to reduced food intake and consequently, weight loss [5]. Further, studies suggest that nicotine's interaction with the central melanocortin system, particularly the melanocortin-4 receptors, also contributes to appetite suppression. Stimulation of these receptors decreases appetite and increases metabolism, further affecting the individual's eating behavior [8, 9]. Smoking has been associated with alterations in metabolism and energy balance. Nicotine increases energy expenditure by raising the resting metabolic rate and promoting lipolysis, the breakdown of fats. This leads to increased fat metabolism and a reduction in body weight [10]. However, nicotine has been found to enhance fat metabolism independent of significant changes in food intake or physical activity, which in turn suggests that nicotine's effects on energy balance are not solely due to decreased appetite but also involve direct metabolic alterations [11]. Ghrelin and leptin are two crucial hormones involved in appetite regulation and energy balance. Their levels are influenced by smoking, which can lead to changes in hunger, satiety and metabolism. Ghrelin is a peptide hormone secreted mainly by the stomach and stimulates appetite by signaling the hypothalamus to induce hunger. It controls metabolism, energy balance, gastrointestinal processes and increases lipogenesis and appetite. Elevated ghrelin levels lead to increased food intake and fat storage [12]. According to some studies in the literature, ghrelin levels in the blood might increase while smoking cigarettes and decline upon quitting the habit [2, 12]. Adipocytes, which are present in white adipose tissue, are the main source of the hormone leptin, which reduces appetite and aids in energy balance regulation. Leptin helps people maintain a healthy weight and regulates hunger. It controls the ratio of the body's stored fat to the amount of food that is consumed in normal conditions. Elevated leptin levels reduce hunger by signaling the brain cells that adipocytes are saturated. It has been shown in previous studies that smoking raises leptin levels, while cessation of smoking lower the levels, whereas, the reverse is also true in few other studies [13].

## Neurochemical reinforcement: role of dopamine in smoking and food consumption:

Dopamine is a key neurotransmitter involved in the reward, motivation and reinforcement of behaviors. Both smoking and eating, particularly highly palatable foods stimulate the dopamine system, reinforcing these behaviors. When individuals quit smoking, the absence of nicotine- induced dopamine release can lead to compensatory behaviors, most commonly increased food intake. Nicotine, the addictive substance in tobacco, activates nicotinic acetylcholine receptors (NACHRS) on dopaminergic neurons in the ventral tegmental area (VTA). This activation increases dopamine release in the nucleus accumbens (NAC), a central part of the brain's reward system, resulting in pleasurable sensations, stress relief and mood enhancement. Repeated nicotine exposure sensitizes the reward system and strengthens associations between environmental cues (like the sight or smell of cigarettes) and dopamine release [14]. Similarly, foods high in sugar, fat and salt also increase dopamine levels in the nucleus accumbens. Similar to nicotine, highly palatable foods stimulate reward centers and reinforce the habit of eating, especially during stress or boredom. Over time, dopaminergic response to food cues becomes conditioned; eventually even the sight, smell, or thought of food can trigger dopamine release and cravings [15]. When individuals quit smoking, they experience a dopamine deficit due to the removal of nicotine stimulation. To compensate, many turn to calorie-dense, palatable foods which can also trigger dopamine release and temporarily restore reward balance. This leads to compensatory eating, which contributes to post-cessation weight gain. This mechanism explains why some smokers hesitate to quit, fearing weight gain as a side effect. Also, some of the smokers develop the habit of consuming sugary food substances or aerated drink soon after the smoking episode. Smoking and sugary substances both stimulate the brain's dopamine system, reinforcing pleasure. Post smoking, the brain is already sensitized due to nicotine-induced dopamine release, making the sweet or fizzy sensation of aerated drinks feel more intense and pleasurable. This combination creates a reinforced habit loop to achieve reward [16].

## Smoking and dietary preferences:

Smoking has been shown to influence dietary preferences and nutritional intake through various mechanisms, including alterations in taste perception, craving for specific types of foods and differences in macronutrient consumption between smokers and non-smokers. Cigarette smoking can impair gustatory sensitivity, leading to diminished taste perception. Studies have found that smokers exhibit significantly decreased taste sensation compared to non- smokers, with higher nicotine dependence correlating with greater taste deficits. Notably, after smoking cessation, taste sensitivity tends to improve over time; approaching levels observed in non-smokers [17]. Additionally, smoking has been associated with decreased sensitivity to sweet. Literature indicates that smokers have a reduced ability to perceive sweetness, which may influence their food preferences and consumption patterns [7]. This alteration in taste perception among smokers may lead to craving for certain types of foods, particularly those high in fat and sugar. The diminished sensitivity to sweetness and other taste might drive smokers to seek more intensely flavored or higher-calorie foods to achieve the desired sensory experience. This compensatory behavior can result in a preference for energy- dense foods, contributing to unhealthy dietary patterns [18]. Further, literature also reveals notable differences in nutrient intake between smokers and non-smokers. Data from the Second National Health and Nutrition Examination Survey revealed that smokers tend to have lower intake of essential nutrients such as vitamin C, folate, fiber and vitamin A compared to non-smokers [19]. In terms of macronutrient consumption, studies have shown that smokers often have higher total caloric intake and consume more carbohydrates than non-smokers. However, these differences in macronutrient intake may be influenced by various factors, including lifestyle and demographic variables [20].

## Smoking, weight control and body composition:

Smoking has complex interactions with body weight and composition, influencing weight regulation through various physiological and behavioral mechanisms. Understanding this relationship is crucial, particularly when considering the effects of smoking cessation on weight and strategies to manage potential weight gain. Nicotine, the primary addictive component in cigarettes, has been shown to suppress appetite and increase metabolic rate, leading to lower body weight in smokers compared to non- smokers. Research indicates that smokers weigh, on average, 4-5 kilograms less than non- smokers. However, smoking is also associated with an unhealthy distribution of body fat, particularly an increase in visceral fat, which elevates the risk of heart disease, stroke and metabolic disorders such as insulin resistance and type 2 diabetes [5]. On the contrary, weight gain is a common concern following smoking cessation. Studies have shown that individuals who quit smoking typically gain between 3 to 9 kilograms within the first eight years of cessation, with most weight gain occurring within the initial six months. Approximately 10% of quitters may experience a weight increase of around 13 kilograms. This post-cessation weight gain can diminish some health benefits of quitting, such as improvements in lung function and may increase the risk of developing type 2 diabetes and hypertension [3]. Moreover, cessation of nicotine intake removes its appetite-suppressing effects, often leading to increased hunger and a preference for high-calorie foods. This compensatory eating behavior contributes significantly to post-cessation weight gain. Furthermore, nicotine also influences the central nervous system by modulating appetite-regulating peptides such as glucagon-like peptide- 1 (GLP-1) and hypocretin-1, which play crucial roles in hunger and satiety signals [21, 22].

## Psychological and behavioral factors:

Smoking and eating behaviors are intricately linked through various psychological and behavioral factors. Various factors like stress, anxiety, social and environmental factors play a significant role in dietary preferences among smokers. Understanding these connections is essential for developing effective interventions to promote healthier lifestyle. Emotional eating refers to the consumption of food in response to negative emotions such as stress, sadness, or anxiety. This behavior can lead to overeating and is associated with the development of eating disorders. Similarly, smoking is often used as a coping mechanism to manage negative emotions. Both behaviors serve as maladaptive strategies to regulate the cause, providing temporary relief but potentially leading to long-term health consequences. Literature indicates that individuals who engage in emotional eating may also be more prone to smoking, as both behaviors are utilized to cope with emotional distress [23]. Stress and anxiety significantly impact both smoking and eating behaviors. Individuals under stress may experience changes in appetite, leading to increased consumption of high-fat, sugary "comfort foods." This response is linked to the release of stress hormones, which can enhance cravings for calorie-dense foods. Similarly, stress can trigger smoking behaviors, as nicotine provides temporary relief from stress and anxiety. The interplay between stress, eating and smoking creates a cycle where each behavior reinforces the other, complicating efforts to adopt healthier habits [24]. For instance, individuals may use smoking as a coping mechanism to manage stress, which in turn can influence their eating behaviors. Literature has shown that smokers may use smoking instead of eating to cope with stress, leading to decreased food intake and body weight. However, after smoking cessation, these individuals may be more susceptible to weight gain when eating is used to cope with stress (Stress eating) [21]. Social and environmental factors significantly influence smoking and eating behaviors. Cultural norms, peer influence and the accessibility of tobacco products and certain foods can encourage the adoption and continuation of these habits. Peer influence significantly has strong associations with both smoking initiation and continuation and food intake across various age groups, especially among adolescents and young adults. This influence can manifest in both healthy and unhealthy dietary behaviors, shaped by social norms, peer modeling and a desire for social acceptance. Previous studies in the literature also indicates that peer behavior is especially noticed in collectivistic cultures and in such societies, individuals are more likely to adopt smoking and food intake pattern that are prevalent within their peer groups [25, 26]. Peer influence can happen by the following mechanism: a) Social modeling, where people tend to mimic the eating behaviors of those around them; b) Perceived social norms, where people are motivated to conform to what they believe others think is appropriate behavior and c) Peer pressure and acceptance, where choosing habits similar to one's peers can be a way to gain or maintain social acceptance [26]. Further, the availability and accessibility of healthy foods are crucial determinants of dietary choices. Individuals usually opt for more readily available unhealthy alternatives during their episode of smoking [27]. Marketing and advertisements of such food substances through media and the socioeconomic status of the individual including income, education and occupation also influence consumer's choice of dietary patterns [28, 29].

## Conclusion:

The intricate relationship between smoking and eating behavior is mediated by a complex interplay of physiological, neurochemical, psychological and environmental factors. Nicotine exerts significant appetite suppressing effects through central pathways on one hand and its cessation often leads to increased hunger and hedonic eating behaviors, on the other. Furthermore, emotional, stress-related, environmental and peer-related factors also significantly shape smoking and dietary habits. Understanding these overlapping behavioral patterns is essential for public health interventions in designing integrated strategies that address both tobacco cessation and nutritional health.
